# Vaccine breakthrough infection leads to distinct profiles of neutralizing antibody responses by SARS-CoV-2 variant

**DOI:** 10.1172/jci.insight.159944

**Published:** 2022-10-10

**Authors:** Michael S. Seaman, Mark J. Siedner, Julie Boucau, Christy L. Lavine, Fadi Ghantous, May Y. Liew, Josh I. Mathews, Arshdeep Singh, Caitlin Marino, James Regan, Rockib Uddin, Manish C. Choudhary, James P. Flynn, Geoffrey Chen, Ashley M. Stuckwisch, Taryn Lipiner, Autumn Kittilson, Meghan Melberg, Rebecca F. Gilbert, Zahra Reynolds, Surabhi L. Iyer, Grace C. Chamberlin, Tammy D. Vyas, Jatin M. Vyas, Marcia B. Goldberg, Jeremy Luban, Jonathan Z. Li, Amy K. Barczak, Jacob E. Lemieux

**Affiliations:** 1 Beth Israel Deaconess Medical Center, Boston, Massachusetts, USA.; 2 Harvard Medical School, Boston, Massachusetts, USA.; 3 Massachusetts General Hospital, Boston, Massachusetts, USA.; 4 Ragon Institute of MGH, MIT and Harvard, Cambridge, Massachusetts, USA.; 5 Brigham and Women’s Hospital Boston, Massachusetts, USA.; 6 Broad Institute, Cambridge, Massachusetts, USA.; 7 UMass Med School, Worcester, Massachusetts, USA.

**Keywords:** COVID-19, Adaptive immunity, Immunoglobulins

## Abstract

Protective immunity against SARS-CoV-2 infection after COVID-19 vaccination may differ by variant. We enrolled vaccinated (*n* = 39) and unvaccinated (*n* = 11) individuals with acute, symptomatic SARS-CoV-2 Delta or Omicron infection and performed SARS-CoV-2 viral load quantification, whole-genome sequencing, and variant-specific antibody characterization at the time of acute illness and convalescence. Viral load at the time of infection was inversely correlated with antibody binding and neutralizing antibody responses. Across all variants tested, convalescent neutralization titers in unvaccinated individuals were markedly lower than in vaccinated individuals. Increases in antibody titers and neutralizing activity occurred at convalescence in a variant-specific manner. For example, among individuals infected with the Delta variant, neutralizing antibody responses were weakest against BA.2, whereas infection with Omicron BA.1 variant generated a broader response against all tested variants, including BA.2.

## Introduction

Breakthrough infection following SARS-CoV-2 vaccination has been observed since the rollout of COVID-19 vaccines (1, 2) and has been associated with specific variants including Beta (3), Delta (4), and Omicron (5). The predisposing factors to breakthrough infection and the consequences of breakthrough infection for SARS-CoV-2 immunity are poorly understood. Both host and viral factors have been implicated. Immunocompromised patients mount poor immune responses after vaccination (6) and are at high risk of infection despite vaccination (7). Furthermore, early studies demonstrated breakthrough infections despite neutralizing activity of patient serum at the time of infection (2). Varying risk of breakthrough infection by SARS-CoV-2 variant suggests that viral factors also play a role (8, 9). Because T cell reactivity to SARS-CoV-2 peptides appears largely preserved across variants (10, 11)— but neutralizing activity of antibodies is markedly decreased to several variants, especially Omicron (12–14) — we hypothesized that (a) antibody-specific responses differ at the time of vaccine breakthrough infection in a variant specific manner and b) immune responses after breakthrough infection shape the immune response to future variants. We enrolled a cohort of ambulatory individuals with symptomatic breakthrough infection and compared the host antibody response at the time of breakthrough infection and after recovery by variant and vaccination status.

## Results

We enrolled 50 ambulatory individuals with symptomatic SARS-CoV-2 infection. The clinical characteristics of this cohort are shown in Table 1. The cohort included individuals infected with Delta (*n =* 19) and Omicron BA.1 (*n =* 31) variants, as well as individuals who were unvaccinated (*n =* 11), vaccinated (*n =* 24), and boosted (*n =* 15). All participants were outpatients, and no patient was hospitalized during observation. We collected a nasal swab and drew blood at the time of acute infection (median 4 days after the onset of symptoms or positive PCR test; range 2–10 days) and again at convalescence (median 17 days after the onset of symptoms or positive PCR; range 14–24 days).

**Figure 1:**
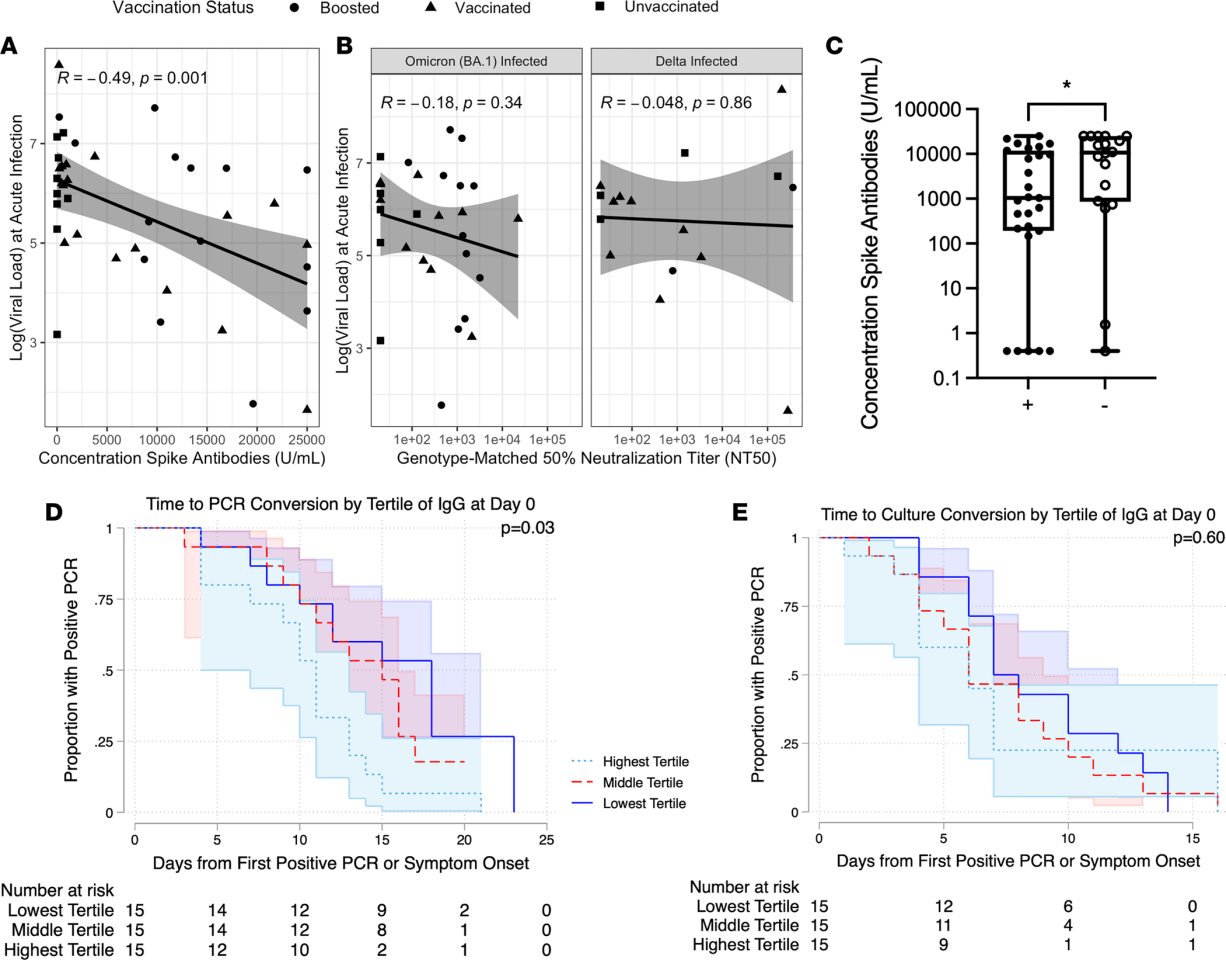
Antibody responses and viral dynamics at the time of acute infection in a breakthrough infection cohort. (**A**) The logarithm of viral load at the time of infection versus the concentration of anti-Spike antibodies at the time of breakthrough. A regression line with standard error is shown, with the Pearson correlation coefficient and corresponding *P* value. (**B**) Genotype-matched neutralizing antibody titers. A regression line with standard error is shown, with the Pearson correlation coefficient and corresponding *P* value. (**C**) Anti-Spike antibody concentration at the initial study visit for culture-positive cases (+) and culture negative cases (–). Significance according to an unpaired Wilcoxon rank-sum test is shown. *P < 0.05. (**D** and **E**) Kaplan-Meier curves for time to PCR conversion by tertile of IgG responses (**D**) and time to culture conversion by tertile of IgG response (**E**). The *P* value represents log-rank testing comparing the subgroups.

We first compared spike-specific antibody levels by vaccination status. All vaccinated patients had high levels of Spike-specific antibodies at the time of breakthrough infection (Supplemental Figure 1; supplemental material available online with this article; https://doi.org/10.1172/jci.insight.159944DS1), with a significant increase in antibody binding titers resulting from breakthrough infection for vaccinated individuals (Supplemental Figure 1; *P <* 0.001, paired Wilcoxon rank-sum test). Nasal viral load at the time of infection correlated inversely with anti-Spike antibody levels at the time of acute infection (Figure 1A; *r* = –0.49, *P =* 0.001). Assessing baseline neutralization activity against individual variants, we found that neutralizing antibody titers against variants also correlated inversely with viral load at the time of breakthrough. This relationship was more variable than for binding antibody titers and was not significant (Figure 1B; *r* = –0.18, *P =* 0.34 for BA.1; *r* = –0.048, *P =* 0.86 for Delta). In addition, the nasal swabs from individuals whose swabs grew in culture had lower levels of anti-Spike antibodies (Figure 1C, P< 0.05 Wilcoxon rank-sum test), and individuals with higher acute anti-Spike antibody titers (by tertile) converted faster to negative PCR (Figure 1D, *P <* 0.05, log rank test). By contrast, we found no relationship between antibody titers at enrollment and time to conversation of viral culture (Figure 1E, *P =* 0.60, log rank test).

We next examined differences in neutralization activity across time points and variants. As expected, neutralizing antibody responses against all the infecting variants increased significantly between acute infection and convalescence (Supplemental Figure 2A and Supplemental Figure 3; Delta-infected against Delta pseudovirus, *P <* 0.001; BA.1-infected against BA.1 pseudovirus, *P <* 0.0001; by paired Wilcoxon rank-sum test). Notably, infection also produced substantial increases in cross-neutralization (Supplemental Figure 2A Supplemental Figure 3; Delta-infected against BA.1 pseudovirus, *P <* 0.01; BA.1-infected against Delta pseudovirus, *P <* 0.001; by paired Wilcoxon rank-sum test). However, this effect was inconsistent: several participants, including 1 vaccinated individual infected with Delta, had no detectable neutralizing activity against BA.1 or BA.2 at convalescence. Overall, BA.2 pseudovirus was the most resistant to neutralization by convalescent serum: among Delta-infected patients at convalescence, there was a 11.2-fold reduction in geometric mean titer against BA.2, compared with Delta. Among BA.1-infected patients at convalescence, there was a 1.8-fold reduction in BA.2 titers as compared with BA.1. Convalescent titers and neutralizing activity were markedly lower for individuals who were unvaccinated at the time of infection, compared with those who had been previously vaccinated or boosted (Supplemental Figure 4). Compared with boosted or vaccinated patients, unvaccinated patients had 38-fold lower neutralization titers against BA.2 (*P <* 0.0001, Wilcoxon rank-sum test), 31-fold lower against BA.1 (*P <* 0.001, Wilcoxon rank-sum test), 33-fold lower against D614G (*P <* 0.01, Wilcoxon rank-sum test), and 25-fold lower (*P <* 0.05, Wilcoxon rank-sum test) against Delta.

**Figure 2:**
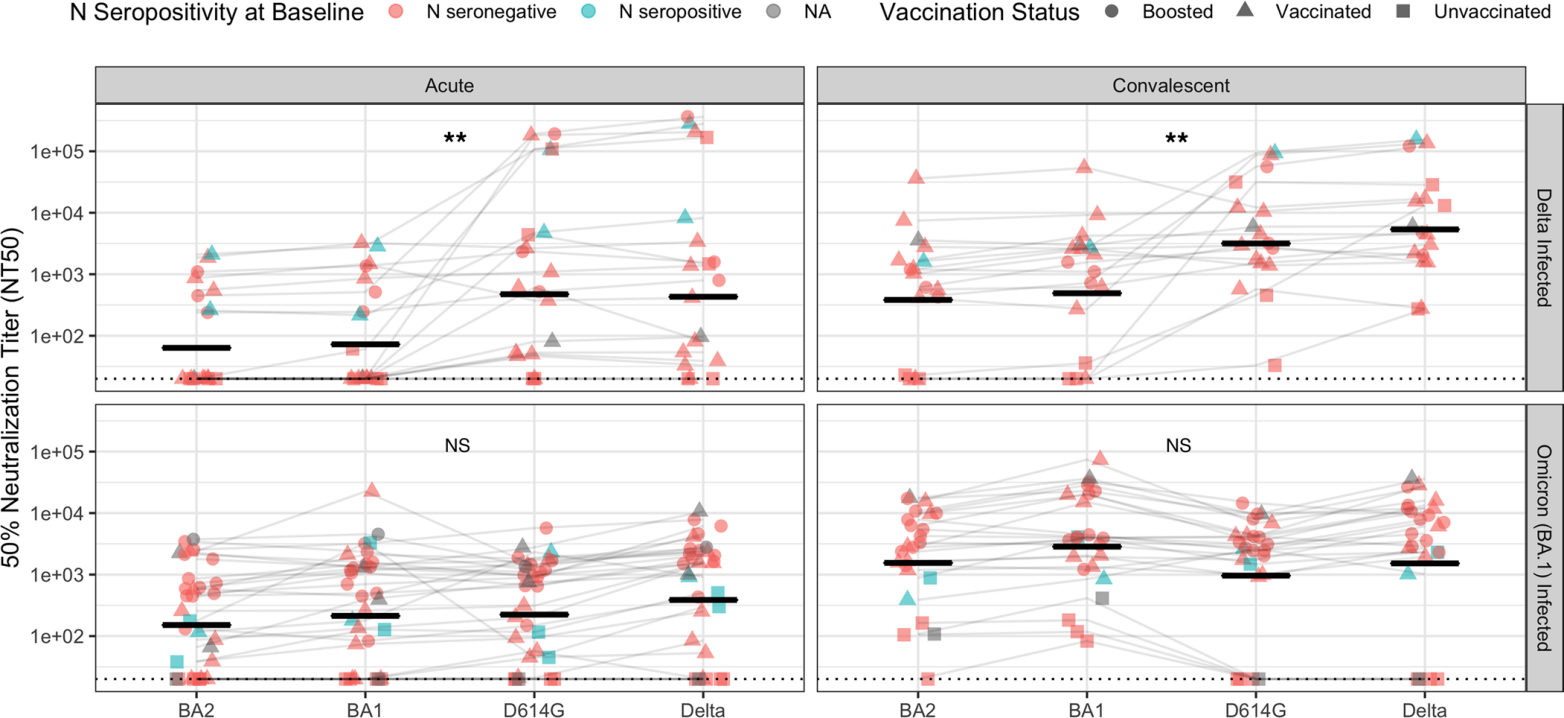
Neutralizing antibody responses, as measured by NT50, against a panel of pseudoviruses. The geometric mean is shown with a bar. Patients infected with Delta variants are shown in the top panel. Patients infected with the Omicron (BA.1) variant are shown in the bottom panel. Significance test of medians (Kruskal-Wallis) is shown. **P < 0.01. The solid bars show geometric mean for each group. Vaccination status is denoted by point shape. Subjects with seronegativity to Nucleocapsid (N) antigens at baseline are colored red; those with N seropositivity are colored blue.

Variant-specific neutralizing activity differed by infecting variant, with Delta-infected patients developing a response that was more specific to WT and Delta variants (Figure 2). Among Delta-infected patients, neutralizing antibody titers differed significantly among variants at both acute (*P <* 0.01, Kruskal-Wallis test) and convalescent time points (*P <* 0.01; Kruskal-Wallis test). In contrast, Omicron-infected patients had neutralizing activity that did not differ significantly among groups (*P >* 0.05 for acute and convalescent time points, Kruskal-Wallis test). Despite a response in Delta-infected individuals that was more specific toward WT and Delta variants, titers in that group still increased against BA.1 and BA.2, indicating an expansion of the antibody repertoire against variants not yet encountered. Convalescent serum from BA.1-infected participants demonstrated significantly higher neutralization titers against BA.1 and BA.2 compared with convalescent serum from Delta-infected participants (Supplemental Figure 2B; *P <* 0.05, *P <* 0.05, Wilcoxon rank-sum test), while maintaining comparable neutralization titers against Delta pseudoviruses (Supplemental Figure 2B).

**Figure 3:**
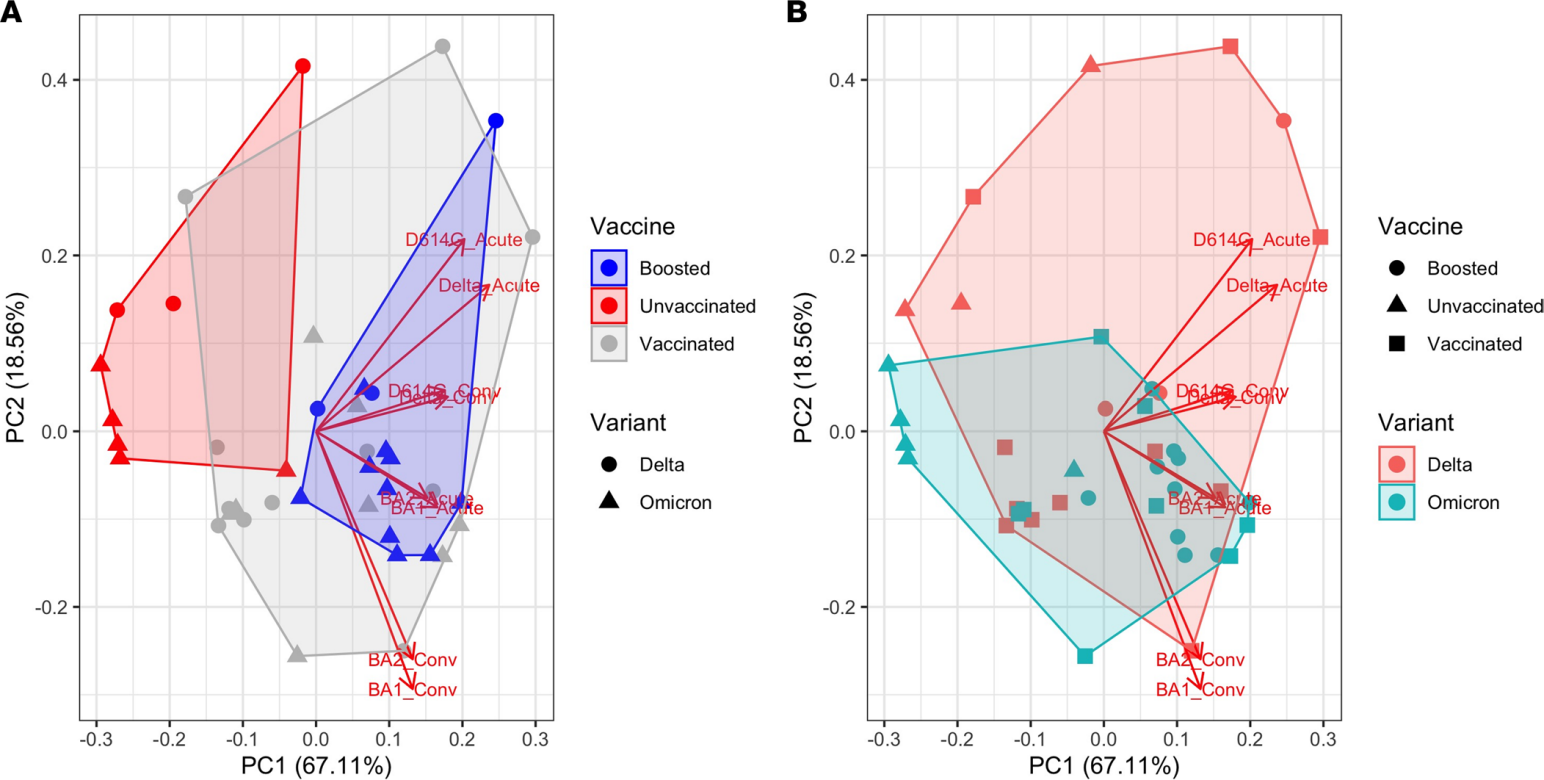
Principal component analysis of NT50 titers. The loadings are plotted as arrows. (**A**) The convex hull of the clusters according to vaccination status is shaded. Point shape denotes the infecting variant. (**B**) The convex hull of the clusters according to the infecting variant is shaded. Point shape denotes vaccination status at the time of infection.

We applied principal component analysis to characterize further the space of neutralizing antibody responses across variants (Figure 3). The first principal component corresponded roughly to strength of immunity, with positive values of the first principal component (PC1, variance explained 67%) corresponding to stronger neutralization responses for all variants tested (Figure 3, variant-specific loadings are plotted as arrows). As a result, unvaccinated cases separated from boosted cases by PC1 alone (Figure 3A). PC2 (variance explained 19%) correlated with the infecting variant, with BA.1-infected cases clustering together. There was substantial overlap in the responses of Delta- and Omicron-infected patients, particularly among those vaccinated and/or boosted, consistent with the broad-neutralizing responses observed for most patients. Taken together, these results suggest that vaccination status is the primary driver of differences in antibody responses across infected individuals and that the infecting variant makes a modest additional contribution.

## Discussion

Breakthrough infection among vaccinated persons is an increasingly common presentation of SARS-CoV-2 infection with novel variants. Previous studies have linked both host and viral factors to the risk of reinfection, but much remains to be learned about the determinants of protection from SARS-CoV-2 infection. Understanding the breadth and potency of immunity against SARS-CoV-2 after vaccination in a variant-specific manner is critical for defining the limitations of the current generation of SARS-CoV-2 vaccines and developing broadly neutralizing vaccine approaches in the future. We show here that antibody binding titers correlate inversely with viral load at the time of breakthrough infection and that antibody titers at infection predict time to PCR conversion but not time to negative viral cultures. Compared with infection with the Delta variant, we found that BA.1 infection induced neutralizing antibodies with greater breadth, including against Delta, BA.1, and BA.2 pseudoviruses. This result is consistent with several other recent studies that have found extremely strong neutralization titers against a panel of variants after breakthrough infection (15–17), with distinct polarization of the immune response as a function of the infecting variant (15).

Neutralization titers have previously been inversely linked to viral load in COVID-19 among unvaccinated patients (18). It is notable that this relationship also holds true in a vaccine-breakthrough cohort. We also show that breakthrough infections among vaccinated patients infected with Delta and Omicron variants induce high-titer neutralizing antibody responses, with the broadest responses being observed after Omicron infection. A subset of Delta-infected individuals showed no neutralizing antibody activity against Omicron variants.

Our results are directly relevant to future SARS-CoV-2 vaccine development. The increased breadth of neutralizing antibody repertoire seen in BA.1-infected, vaccinated individuals compared with Delta-infected, vaccinated individuals suggests that booster regimens that span the antigenic landscape of circulating SARS-CoV-2 genetic variation may have potential as one component of a future vaccination strategy to elicit broadly neutralizing antibody responses. Observations of responses in humans naturally infected with different variants complement controlled studies in animal models. Although infection is not directly comparable with vaccination, our results are consistent with the observed enhancement of neutralization activity after boosting an mRNA-1273 primary series with mRNA-1273-Omicron (19). Nonhuman primate studies of the mRNA-1273 booster, however, show no differences in neutralization response (20). Defining the strategies that elicit durable immunity against current and future variants is an important area of SARS-CoV-2 research.

Our findings are also potentially relevant to SARS-CoV-2 epidemiology. The Omicron variant has overtaken the globe faster than any previous SARS-CoV-2 variant (21). Its increased fitness appears linked, at least in part, to its extensive antibody evasion properties and ability to cause vaccine breakthrough infections (22) and reinfections (5). Initially, most Omicron cases were caused by BA.1, but the BA.2 variant appears to have a higher growth rate in most populations and is replacing BA.1 as the dominant variant in many locations (23, 24). Among the variants tested here, neutralization titers were lowest against BA.2. After BA.1 infection, we found a modest 1.8-fold decrease in geometric mean in BA.2 neutralization compared with BA.1 neutralization, roughly concordant with other recently reported data (25, 26). Nevertheless, because estimates are consistent in showing a 30%–40% growth advantage per viral generation of BA.2 over BA.1 (23, 24), and BA.1 appears to derive much of its fitness advantage from antibody escape (23), these modest differences in neutralization titers may be epidemiologically significant. As the emergence of new variants continues — with BA.1 replaced by BA.2, which has been since been replaced by its sublineages BA.2.12.1 and BA.4/5 — it is important to characterize the cross-neutralization properties of these variants in unvaccinated, vaccinated, and hybrid immune populations, particularly as the current phase of the pandemic appears to be driven by antibody escape (21, 23, 27, 28).

Among vaccinated persons, “breakthrough” infections are not unexpected; the primary goal of vaccination has been protection against severe disease rather than blocking transmission. The weak neutralizing antibody responses following infection in unvaccinated persons suggests that convalescent immunity alone, in the absence of vaccination, is unlikely to be sufficient to afford protection against future variants. In summary, our results highlight the increase in neutralizing activity following variant-specific breakthrough infection and emphasize its breadth across variants, particularly among vaccinated persons infected with Omicron BA.1. However, our findings also suggest that broad swaths of the population remain susceptible to circulating variants and underscore the importance of vaccination efforts.

## Methods

### Study recruitment.

Adult outpatients diagnosed with COVID-19 were recruited as soon as possible after a positive SARS-CoV-2 test. Although symptomatic disease was not a requirement for enrollment, all but 1 patient was symptomatic at the time of diagnosis; the 1 asymptomatic patient was diagnosed at screening for work/travel. Phlebotomy was performed at the initial visit and at subsequent visit approximately 14 days later. Anterior nasal swabs were collected 3 times weekly at home visits until negative PCR testing and stored in viral transport medium. Specimens were transported to the laboratory within 4 hours of collection. Viral transport medium containing anterior nasal swabs was aliquoted and stored at –80°C until testing. To isolate serum, blood was collected into BD red-top (anticoagulant-free) vacutainer tubes and processed according to manufacturer instructions. Serum was stored in aliquots at –80°C until processing.

### Laboratory methods.

Viral genotype was determined using Spike gene sequencing and whole-genome sequencing as previously described (29). Viral load quantification and viral culture were also performed as previously described (29). Sequence data were submitted to Genbank under accession no. PRJNA759255. ELISA assays were performed using the Elecsys Anti-SARS-CoV-2 ELISA assays for Spike and Nucleocapsid antibodies as per manufacturer instructions (presumed WT). Concentrations exceeding 25,000 U/mL were set to the assay maximum of 25,000, and nonparametric tests in paired comparisons were used to account for this. Pseudovirus neutralization assays were performed as previously described (30). Nucleocapsid antibodies were considered as “positive” (suggestive of prior infection) with a threshold of 0.8U/mL. Neutralizing activity against SARS-CoV-2 pseudovirus was measured using a single-round infection assay in 293T/ACE2 target cells. Pseudotyped virus particles were produced in 293T/17 cells (ATCC) by cotransfection of plasmids encoding codon-optimized SARS-CoV-2 full-length Spike (containing G at position 614), packaging plasmid pCMV DR8.2, and luciferase reporter plasmid pHR’ CMV-Luc. WT, Omicron, BA.1 and BA.2 Spikes, packaging, and luciferase plasmids were provided by Nicole Doria Rose (NIH Vaccine Research Center, Bethesda, Maryland, USA). Delta variant Spike plasmid was provided by Bing Chen (Children’s Hospital, Boston, Massachusetts, USA). The 293T cell line stably overexpressing the human ACE2 cell surface receptor protein was provided by Michael Farzan (University of Florida Scripps Biomedical Research Institute, Jupiter, Florida, USA) and Huihui Ma (The Scripps Research Institute, La Jolla, California, USA). For neutralization assays, serial dilutions of patient serum samples were performed in duplicate, followed by addition of pseudovirus. Pooled serum samples from convalescent COVID-19 patients or prepandemic normal healthy serum (NHS) were used as positive and negative controls, respectively. Plates were incubated for 1 hour at 37°C, followed by addition of 293/ACE2 target cells (1 × 10^4^/well). Wells containing cells + pseudovirus (without sample) or cells alone acted as positive and negative infection controls, respectively. Assays were harvested on day 3 using Promega BrightGlo luciferase reagent, and luminescence was detected with a Promega GloMax luminometer. Titers are reported as the dilution of serum that inhibited 50% or 80% virus infection (ID_50_ and ID_80_ titers, respectively). Serum samples were tested in duplicate using a primary 1:20 dilution with 3-fold or 5-fold titration series. We defined vaccinated patients as having completed a full primary immunization series, either 2 doses of mRNA-1273 or BNT162B2 or a single dose of Ad26.COV2.S at least 14 days prior to enrollment. We defined boosted patients as having completed a third dose of either mRNA-1273 or BNT162B2 at least 14 days prior to enrollment.

### Statistics.

Statistical analysis was performed using Stata version 16.1 (Statacorp) and R v4.0.1 (31) and visualized using ggplot2 (32) and the ggpubr package. Wilcoxon rank-sum tests were used to compare the medians between 2 groups. Kruskal-Wallis tests were used to compare the medians of multiple groups. For survival analysis, we used the Kaplan-Meyer method to estimate the survival function for time to conversion of negative PCR and viral culture, by tertile of serologic titers against Spike protein at enrollment. *P* < 0.05 was considered statistically significant.

### Study approval.

Study procedures were reviewed and approved by the Human Subjects IRB and the Institutional Biosafety Committee at Mass General Brigham under protocol no. 2021P000812. All participants gave verbal informed consent, as written consent was waived by the review committee based on the risk/benefit ratio of requiring in-person interactions for an observational study of COVID-19.

## Author contributions

MSS conceived of the study design, participated in the data analysis, and contributed editorial input. MJS conceived of the study design, participated in the data analysis, and contributed editorial input. JB conceived of the study design, conducted experiments, participated in the data analysis, and contributed editorial input. CLL conducted experiments, participated in the data analysis, and contributed editorial input. FG conducted experiments, participated in the data analysis, and contributed editorial input. MYL participated in the data collection, conducted experiments, participated in data analysis, and contributed editorial input. JIM participated in the data collection, conducted experiments, participated in data analysis, and contributed editorial input. AS participated in the data collection and contributed editorial input. CM participated in the data collection, conducted experiments, and contributed editorial input. JR participated in the data collection, conducted experiments, and contributed editorial input. RU participated in the data collection, conducted experiments, participated in data analysis, and contributed editorial input. MCC conducted experiments, participated in the data analysis, and contributed editorial input. JPF conducted experiments and contributed editorial input. GC participated in data collection and contributed editorial input. AMS participated in data collection and contributed editorial input. TL participated in data collection and contributed editorial input. AK conducted experiments, participated in the data analysis, and contributed editorial input. MM conducted experiments, participated in the data analysis, and contributed editorial input.RFG directed the data collection, participated in the data analysis, and contributed editorial input. ZR directed the data collection, participated in the data analysis, and contributed editorial input. SLI participated in data collection and contributed editorial input. GCC participated in data collection and contributed editorial input. TDV participated in the data collection and contributed editorial input. JMV conceived of the study design, provided reagents, and contributed editorial input. MBG conceived of the study design, provided reagents, participated in the data analysis, and contributed editorial input. JL conceived of the study design, provided reagents, participated in the data analysis, and contributed editorial input. JZL conceived of the study design, provided reagents, participated in the data analysis, and contributed editorial input. AKB conceived of the study design, provided reagents, participated in the data analysis, and contributed editorial input. JEL conceived of the study design, provided reagents, participated in the data analysis, drafted the initial manuscript, and contributed editorial input. All authors approve of the final version of the manuscript. Order of co–first and co–senior authors was determined by rotation due to an ongoing collaboration among the investigators.

## Supplementary Material

Supplemental data

## Figures and Tables

**Table 1:**
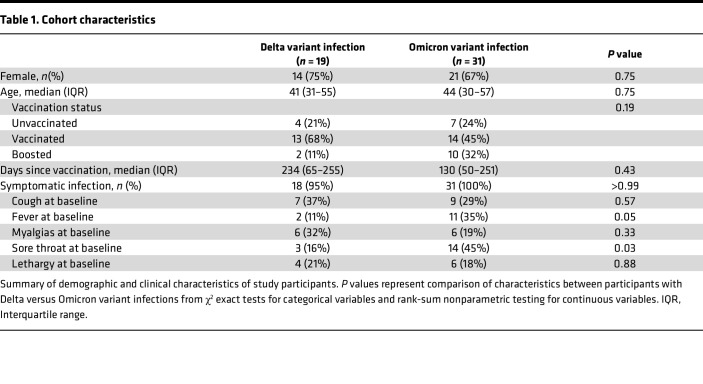
Cohort characteristics

## Version 1 10/10/2022

Electronic publication
