# Supplementary material for: Vaccine breakthrough infection leads to distinct profiles of neutralizing antibody responses by SARS-CoV-2 variant
Source: JCI Insight. 2022 Oct 10;7(19):e159944. doi: 10.1172/jci.insight.159944 (PMC9675445; doi:10.1172/jci.insight.159944)
Supplement: Supplemental data [file jciinsight-7-159944-s255.pdf]

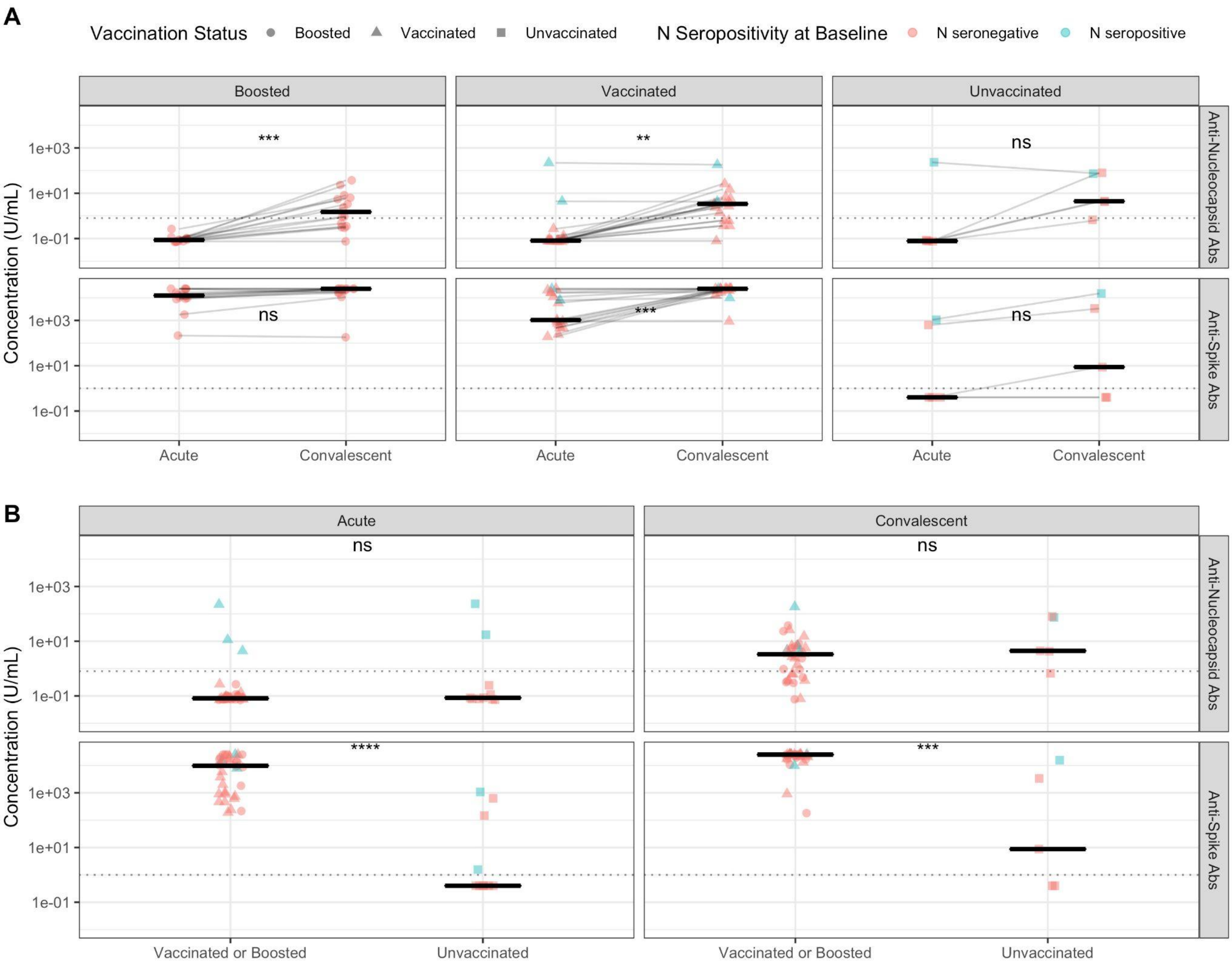

Figure S1: **A.** Antibody titers as measured by ELISA against Spike and Nucleocapsid protein at the time of acute infection and convalescence. Significance from a paired Wilcoxon test is shown. **B.** Antibody titers against Spike and Nucleocapsid protein by vaccination status. Significance from an unpaired Wilcoxon test is shown. ns: $p > 0.05$; * - $p < 0.05$; *** - $p < 0.001$; **** - $p < 0.0001$. The solid bars show geometric mean for each group. The dashed line indicates the manufacturer-defined threshold for positivity (0.8U/mL for N, 1.0U/mL for Spike).

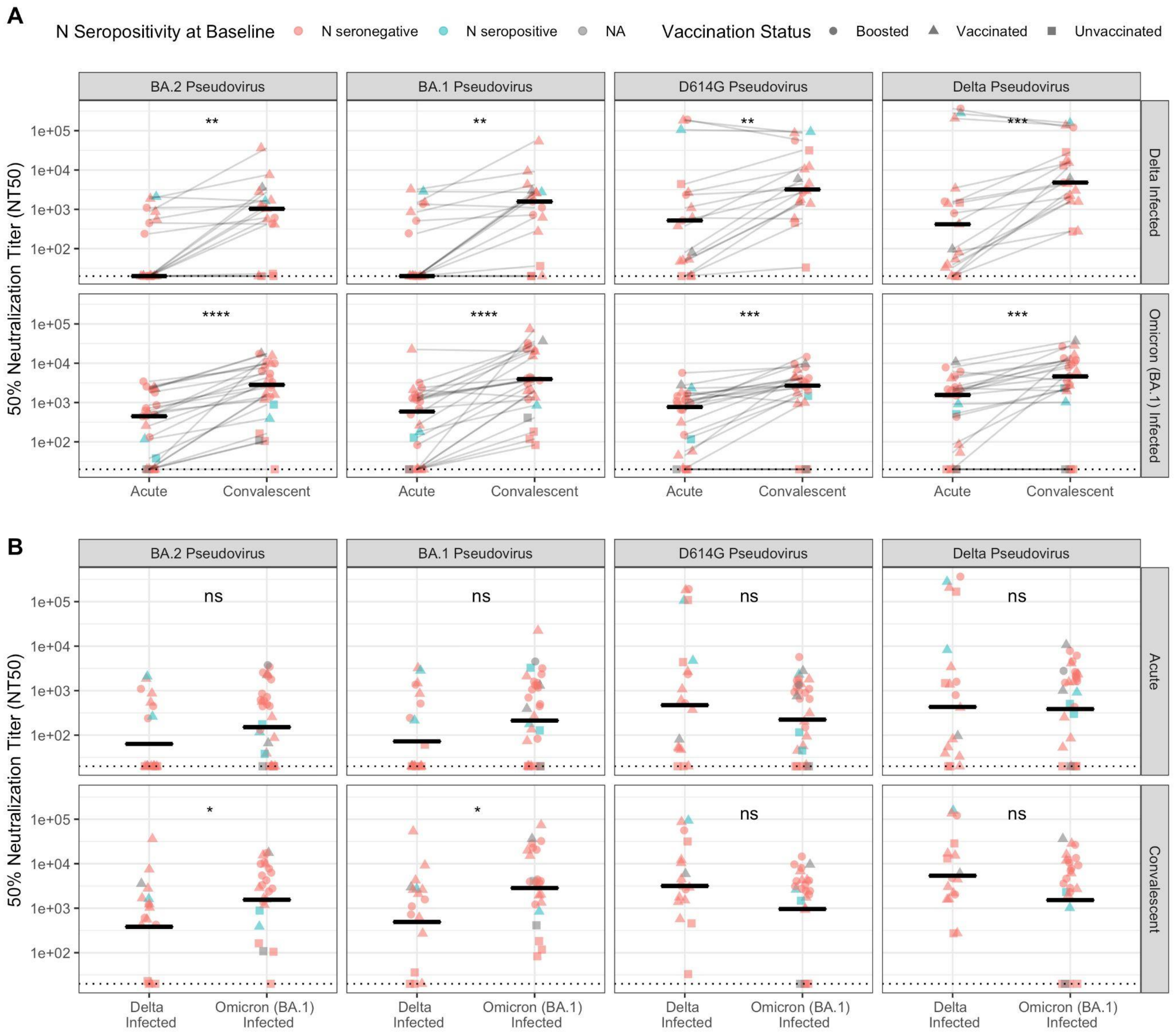

Figure S2: A. Neutralizing antibody responses against a panel of pseudoviruses, as measured by the 50% neutralizing antibody titer (NT50), at the time of acute infection and convalescence against a panel of pseudoviruses. Significance from a paired Wilcoxon test is shown. **B.** Neutralizing antibody responses by the infecting variant. Significance from an unpaired Wilcoxon test is shown. ns - p > 0.05; * - p < 0.05; ** - p < 0.01; *** - p < 0.001; **** - p < 0.0001. The solid bars show geometric mean for each group. The dashed line denotes the limit of detection of the assay (serum ID50 titer 1:20).

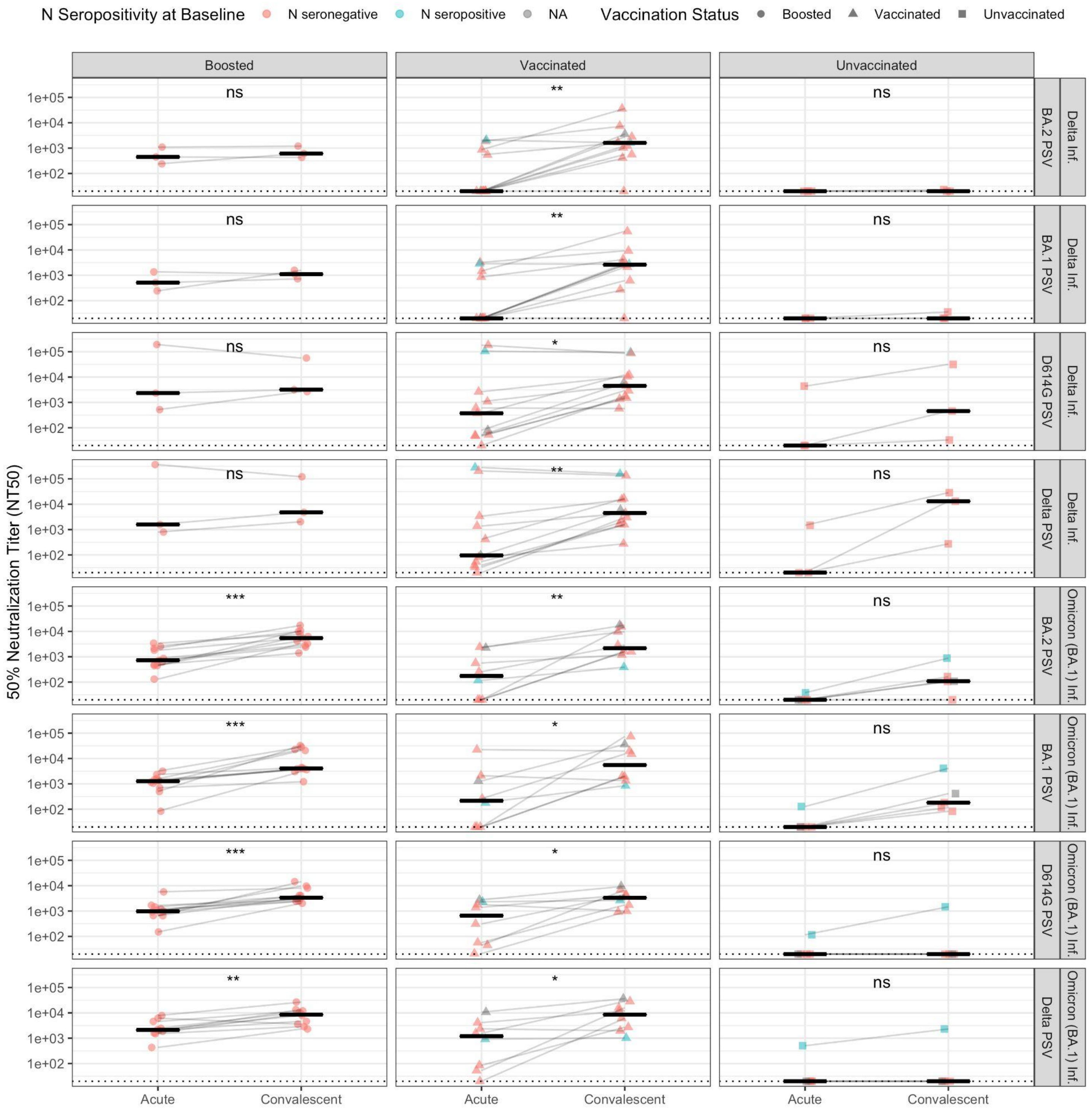

Figure S3: Neutralizing antibody responses, as measured by the 50% neutralizing antibody titer (NT50), at the time of acute infection and convalescence against a panel of pseudoviruses. Responses are stratified by the genotype of the infecting variant, the genotype of the pseudovirus, and the vaccination status of the individual. Significance from a paired Wilcoxon test is shown. ns: $p > 0.05$; * - $p < 0.05$; ** - $p < 0.01$; *** - $p < 0.001$. The solid bars show geometric mean for each group. The dashed line denotes the limit of detection of the assay (serum ID50 titer 1:20).

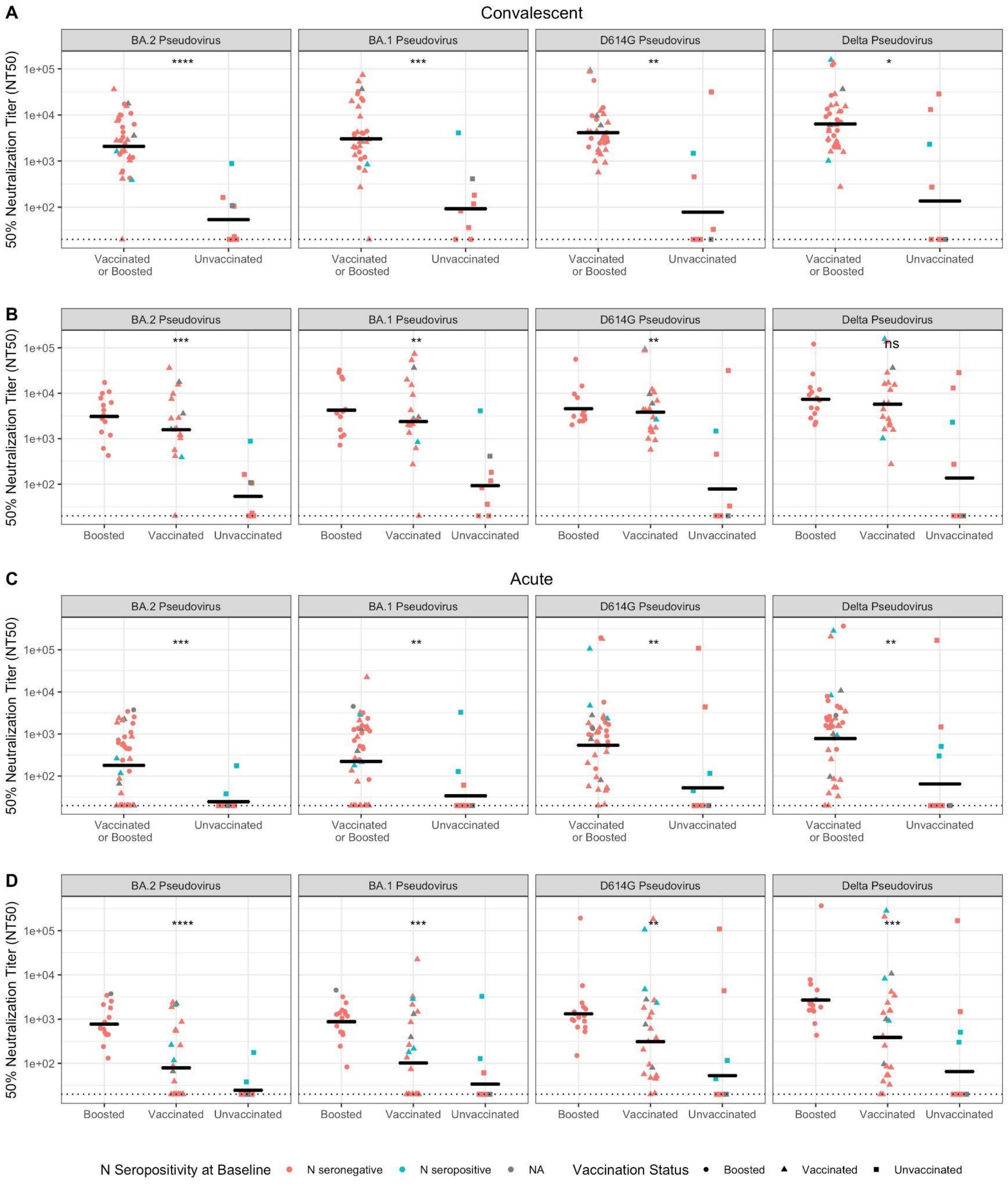
A
Convalescent
B
C
Acute
D
BA.2 Pseudovirus
BA.1 Pseudovirus
D614G Pseudovirus
Delta Pseudovirus
50% Neutralization Titer (NT50)
1e+02
1e+03
1e+04
1e+05
Vaccinated or Boosted
Unvaccinated
Boosted
Vaccinated
****
***
**
*
ns
N Seropositivity at Baseline
N seronegative
N seropositive
NA
Vaccination Status
Boosted
Vaccinated
Unvaccinated

Figure S4: A. Neutralizing antibody responses, as measured by the 50% neutralizing antibody titer (NT50), at the time of convalescence, in unvaccinated, vaccinated or boosted individuals, against a panel of pseudoviruses. Significance from a Wilcoxon rank-sum test is shown. **B.** Neutralizing antibody responses, as measured by the 50% neutralizing antibody titer (NT50), at the time of convalescence, in unvaccinated, vaccinated, or boosted individuals, against a panel of pseudoviruses. **C.** Neutralizing antibody responses, as measured by the 50% neutralizing antibody titer (NT50), at the time of acute infection, in unvaccinated, vaccinated or boosted individuals, against a panel of pseudoviruses. Significance from a Wilcoxon rank-sum test is shown. **D.** Neutralizing antibody responses, as measured by the 50% neutralizing antibody titer (NT50), at the time of acute infection, in unvaccinated, vaccinated, or boosted individuals, against a panel of pseudoviruses.Significance from a Kruskal-Wallis test is shown. ns: $p > 0.05$; * - $p < 0.05$; ** - $p < 0.01$; *** - $p < 0.001$; **** - $p < 0.0001$. The solid bars show geometric mean for each group. The dashed line denotes the limit of detection of the assay (serum ID50 titer 1:20).
